# Estimation of population age structure, daily survival rates, and potential to support dengue virus transmission for Florida Keys *Aedes aegypti* via transcriptional profiling

**DOI:** 10.1371/journal.pntd.0012350

**Published:** 2024-08-13

**Authors:** Catherine A. Pruszynski, Eva A. Buckner, Nathan D. Burkett-Cadena, Leon E. Hugo, Andrea L. Leal, Eric P. Caragata

**Affiliations:** 1 Florida Keys Mosquito Control District, Marathon, Florida, United States of America; 2 University of Florida, Institute of Food and Agricultural Sciences, Department of Entomology and Nematology, Florida Medical Entomology Laboratory, Vero Beach, Florida, United States of America; 3 Mosquito Control Laboratory, QIMR Berghofer Medical Research Institute, Brisbane, Queensland, Australia; University of California Davis School of Veterinary Medicine, UNITED STATES OF AMERICA

## Abstract

*Aedes aegypti* is an important vector of dengue virus and other arboviruses that affect human health. After being ingested in an infectious bloodmeal, but before being transmitted from mosquito to human, dengue virus must disseminate from the vector midgut into the hemocoel and then the salivary glands. This process, the extrinsic incubation period, typically takes 6–14 days. Since older mosquitoes are responsible for transmission, understanding the age structure of vector populations is important. Transcriptional profiling can facilitate predictions of the age structures of mosquito populations, critical for estimating their potential for pathogen transmission. In this study, we utilized a two-gene transcript model to assess the age structure and daily survival rates of three populations (Key West, Marathon, and Key Largo) of *Ae*. *aegypti* from the Florida Keys, United States, where repeated outbreaks of autochthonous dengue transmission have recently occurred. We found that Key Largo had the youngest *Ae*. *aegypti* population with the lowest daily survival rate, while Key West had the oldest population and highest survival rate. Across sites, 22.67% of *Ae*. *aegypti* females were likely old enough to transmit dengue virus (at least 15 days post emergence). Computed estimates of the daily survival rate (0.8364 using loglinear and 0.8660 using non-linear regression), indicate that dengue vectors in the region experienced relatively low daily mortality. Collectively, our data suggest that *Ae*. *aegypti* populations across the Florida Keys harbor large numbers of older individuals, which likely contributes to the high risk of dengue transmission in the area.

## Author summary

Within a mosquito population it is generally only the very old mosquitoes that are capable of spreading disease-causing pathogens, like dengue virus. Scientists have been developing fast, accurate, and cheap methods to estimate mosquito age. In this study, we used one of those methods to measure the activity of age-responsive genes in dengue virus-transmitting *Aedes aegypti* mosquitoes from the Florida Keys, a dengue hotspot in the United States. This method allowed us to estimate the percentage of older mosquitoes in this region. We found that a high percentage of *Ae*. *aegypti* mosquitoes in Key Largo, Marathon, and Key West (between 11–32%) were likely old enough to be able to spread dengue virus. We also observed that *Ae*. *aegypti* from the Florida Keys experienced relatively low daily mortality, as the daily probability of survival for any given mosquito was estimated at 86–88%. These findings suggest that conditions in the Florida Keys are very suitable for mosquitoes to thrive, reach old age, and potentially contribute to dengue outbreaks in the region.

## Introduction

The mosquito *Aedes aegypti* (Linnaeus) is the principal vector of yellow fever, chikungunya, Zika, and dengue viruses in tropical and subtropical regions of the world. Dengue is a reemerging disease that has seen a rapid increase in transmission and expansion into many tropical and sub-tropical locations in the last few decades. All four dengue serotypes are now found in the Americas, which is problematic given that previous exposure to one of the four dengue serotypes has been shown to increase the risk of more severe symptoms during a subsequent infection with a different serotype [1,2]. In the continental United States, dengue outbreaks involving locally acquired cases have occurred in Arizona, California, Florida and Texas in the past two decades, with most cases occurring in Florida [3].

Vectorial capacity for a mosquito population is an estimate of the transmission efficiency of vector-borne diseases, such as dengue, and is influenced by mosquito population size, human biting rate, vector competence, the extrinsic incubation period (EIP), and the daily probability of mosquito survival (*p*) [4,5]. Survival rates vary depending on mosquito species but are typically in the range of 0.7 to 0.9, meaning the daily mortality rate is approximately 10–30% [6]. Historically, *p* has been estimated using a range of different linear or non-linear hazard functions that incorporate parameters inferred from longevity or mark-release-recapture experiments [7–9].

Mosquito longevity and *p* are critical determinants of the transmission potential of a mosquito population because they impact overall reproduction rates as well as its capacity to transmit a pathogen [10,11]. However, both parameters are difficult to estimate for wild mosquitoes because it is difficult to accurately estimate mosquito age. The length of time before a mosquito can transmit a virus is closely linked to mosquito longevity and the EIP, as mosquito-borne viruses, like dengue, require a period of between 6–14 days after they are ingested in an infectious blood meal before they can be passed to a new host during subsequent feeding [12]. As such, it is only the cohort of older mosquitoes in a population, which have lived beyond the minimum EIP that are responsible for dengue transmission.

Accordingly, a potentially tractable goal of vector control is preventing mosquito vectors from living long enough to transmit pathogens, as opposed to eliminating vector populations altogether. This means that a method of accurately assessing changes to a mosquito population age structure is a critical tool to evaluate the success of vector control interventions. This is important when interventions differentially impact mosquitoes according to age, as is the case with some insecticides [13]. Development of accurate methods of age determination (also known as age grading) is therefore essential to gain insight into mosquito populations, vector control interventions, and vectorial capacity.

Historical methods of age prediction dissections of mosquitoes to observe ovaries to determine if a female was nulliparous, and therefore relatively young [14], and ovariolar dilatations to estimate the number of gonotrophic cycles [15] lose accuracy beyond their first gonotrophic cycle [16] and therefore their use in age grading can lead to overestimation of the number of post-EIP mosquitoes in a population [17]. Therefore, precise, inexpensive, and simple methods of age determination for mosquitoes are needed. Several novel approaches to age-grading utilized in other insects have been applied to mosquitoes with varying degrees of success. For example, measuring pteridine concentrations is effective for some fly species but not for *Anopheles albimanus* (Wiedemann), *Aedes polynesiensis* (Marks), or *Culex quinquefasciatus* (Say) [18,19]. Approaches such as gas-liquid chromatography-based profiling of cuticular hydrocarbons in *Ae*. *aegypti* requires specialized equipment and technical expertise but can also be unreliable for some mosquito species and require very large sample sizes [20–22]. Near- and Mid-infrared spectroscopy approaches to age grading have recently been applied. These techniques measure the absorption of age-dependent organic compounds in a mosquito sample, and are promising, but require the use of highly specialized spectrometers and results from field trials have been mixed [23–26].

Age determination through transcriptional profiling uses Reverse Transcriptase quantitative Polymerase Chain Reaction (RT-qPCR) to estimate mosquito age via quantification of gene transcripts that exhibit age-dependent changes in expression. Expression profiles of these genes can be compared between mosquito populations of known age reared under laboratory or semi-field or field-conditions and mosquitoes of unknown age collected from the field [27]. Multiple studies have demonstrated the functionality of this approach with estimates of age made to within a few days of actual age [17,28–33]. While transcriptional profiling requires proficiency of multiple molecular techniques and can have a high cost per sample, costs and time spent on analysis can potentially be decreased by reducing the number of genes used to generate the age prediction model [28,32]. We selected transcriptional profiling as our age estimation method because many mosquito control districts and public health laboratories already have RT-qPCR capabilities for pathogen detection, and this approach utilizes the same equipment and skillset.

Dengue case rates in the United States are likely to increase in regions where *Ae*. *aegypti* is present due to frequent introductions of the virus associated with international travel [34]. In the Florida Keys, an autochthonous dengue outbreak occurred in Key West between 2009 to 2010 [35] with a subsequent outbreak occurring in Key Largo during the course of this study [36]. The inhabited areas within the Keys all support persistent *Ae*. *aegypti* populations. However, it is unclear whether these populations have similar age structures or experience similar rates of daily survival. Differences in mosquito survival result in geometric changes to mosquito vectorial capacity and will shape the likelihood of dengue outbreaks across the Keys. To improve understanding of the capacity of the *Ae*. *aegypti* populations in this region to support dengue transmission, we used age grading via transcriptional profiling to estimate the age structures of *Ae*. *aegypti* populations from three locations in the Florida Keys: Key West, Marathon, and Key Largo. We then used those data to calculate the daily probability of survival for *Ae*. *aegypti* mosquitoes in the region using loglinear or non-linear methods, which assumed either constant or variable survival rates with age, respectively.

## Methods

### Study locations

**Fig 1:**
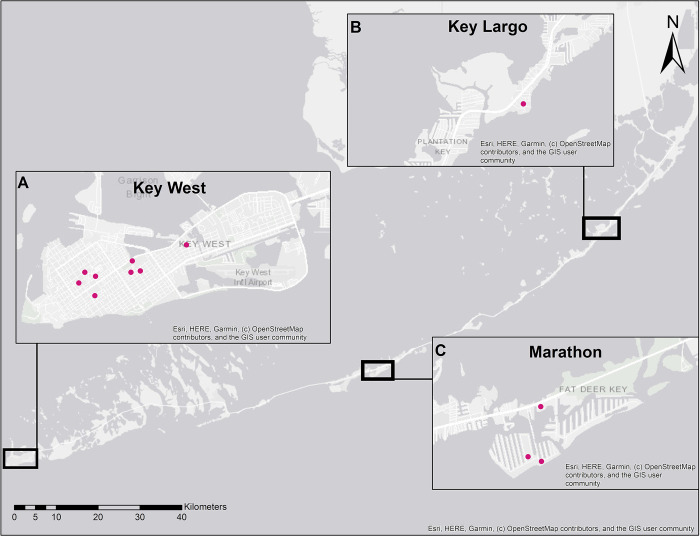
Map of the Florida Keys highlighting *Aedes aegypti* collection sites. Adult *Aedes aegypti* specimens used for age grading were collected from three locations in the Florida Keys, Key West (A), Key Largo (B), and Marathon (C) between October and December 2020 using BG Sentinel traps. Pink dots represent trap locations. Scale does not refer to map insets. Map created using ArcGIS software by ESRI (ESRI, Redlands, CA). Base map was sourced by ESRI through ArcGIS Online.

Female *Ae*. *aegypti* mosquitoes used in this project were collected from three locations in the Florida Keys: Key West, Marathon, and Key Largo (Fig 1). Key West (24.555°, 81.7825°) is the southernmost incorporated city of Florida and covers an area of 10.9 km^2^. It is a densely populated urban area with 26,444 residents [37]. Marathon (24.7260° N, 81.0446° W) is located approximately 80 km northeast of Key West and has an area of 19.17 km^2^. Its population of 9,689 is spread over seven small suburban and urban islands [38]. Key Largo (25.086° N, 80.4473° W) is 80 km northeast of Marathon and 160 km northeast of Key West and has an area of 39.49 km^2^ and a population of 12,447 [39]. All three areas have a tropical savanna climate (Köppen-Geiger climate classification Aw/As) with wet and dry seasons of approximately equal duration [40]. Mosquito collections, detailed below, were performed between November and December 2020. Temperature and precipitation measurements during the collection period were obtained from the National Weather Service weather stations at the Key West Airport (Key West), Curry Hammock State Park (Marathon), and John Pennekamp State Park (Key Largo). Mean daily temperature and precipitation data were compared between the three sites using One-way Analysis of Variance (ANOVA) using Minitab statistical software (Minitab LLC, USA).

### Known age mosquito samples

Progeny of *Ae*. *aegypti* adults reared from wild larvae from Key Largo, FL served as material for a “known age” colony of mosquitoes that was used to calibrate and validate the age prediction model. The larvae were collected from residential properties and identified to species by Florida Keys Mosquito Control District (FKMCD) employees during routine mosquito inspections in Key Largo during September 2020. Approximately 100 of these larvae were reared to adulthood in a 27 cm^3^ cage in an environmental chamber in the FKMCD laboratory in Marathon, Florida, which was set at 28°C with 80 ± % RH and a 12:12 h L:D cycle. Larvae were fed a small pinch of Tetramin fish flakes ground to powder using a mortar and pestle (Tetra GmBH, Germany), and once emerged, adults were fed 10% sugar solution, *ad libitum*, via cotton wicks. Every three days, adults were offered the chance to feed on defibrinated bovine blood (Hemostat Labs, Dixon, CA, USA) in a tied-off intestine filled with blood, which was heated by exposure to warm water and then placed in the cage. A 500 mL black, plastic cup containing 300 mL of spring water (Zephyrhills, Zephyrhills, FL, USA) and seed germination paper (Anchor Paper Company, St. Paul MN, USA) was used as an oviposition site. Egg papers were removed and replaced with fresh paper every week for 4 weeks in order to facilitate collection of large numbers of F_1_ eggs.

Approximately 1500 F_1_ generation eggs were hatched synchronously under vacuum for 30 minutes. Larvae were separated into groups of 100 first instars in 200 mL polystyrene cups containing 100 mL of spring water and were fed 10 mg of a 1:1 mix of Brewer’s yeast and liver powder, once daily. This was increased to 20 mg per day for third and fourth instar larvae. Larval cups were kept under ambient environmental conditions in a covered, open-air porch located on Big Pine Key, FL (a suburban island located halfway between Marathon and Key West) during October and November 2020. Pupae were separated from larvae and moved to new polystyrene cups, which were placed in plastic and wire mesh (40x40x40 cm) cages. Adults that eclosed within a 24-hour period were considered to be in the same cohort and transferred to their own cage. A total of three cages each containing approximately 300 adult mosquitoes were produced. Five adult females were removed from each cage every four days between the first and 29^th^ days post-eclosion (dpe) following established protocols for transcriptional profiling of mosquitoes [27]. During collections, mosquitoes were anesthetized with carbon dioxide, then legs, wings, and abdomens were removed with sterile forceps, while heads and thoraces were placed in individual 2.0 mL microcentrifuge tubes containing 300 μL of RNA*later* (Thermofisher Scientific, USA), which were stored in a -80°C freezer.

### Field-captured *Aedes aegypti* of unknown age

Wild *Ae*. *aegypti* females were collected from Key West, Marathon, and Key Largo between October and December 2020 to provide “test” mosquito cohorts of unknown age (S1 File). The collection period overlapped with the known-age assay in order to reduce the potential impact of environmental variation on gene expression. BG Sentinel traps (Biogents, Germany) were used to collect adult host-seeking females from twelve different sites on residential properties across the three locations. Traps were baited with 1.4 kg of dry ice and BG Lures, then deployed at 14:00 before being collected at 10:00 the following day. BG catch bags were returned to the lab and mosquitoes were briefly anesthetized with carbon dioxide to immobilize them. Non-blood fed *Ae*. *aegypti* females were retained, dissected, and stored at -80°C, as above.

### Transcriptional profiling

#### RNA isolation

Individual head/thorax samples from known and unknown age specimens were removed from RNA*later* and transferred to a new sterile, RNase-free, 1.5 mL microcentrifuge tubes containing 200 μL of Trizol (Invitrogen, USA). Samples were manually homogenized with a sterile pestle for one minute. RNA was then extracted from each sample using the TRIzol protocol for RNA isolation from small tissues with isopropanol precipitation performed overnight in a -20°C freezer to increase RNA yield [41]. RNA was rehydrated in 20 μL of RNase-free water. A Nanodrop spectrophotometer (Thermofisher Scientific, USA) was used to evaluate the quality and concentration of RNA for each sample. As an extraction quality benchmark, only samples that had an A_260/280_ ratio above 1.68 were used in downstream applications.

#### DNase treatment and cDNA synthesis

A standardized quantity of 500 ng of total RNA per sample was used for first strand cDNA synthesis. Genomic DNA was removed using the DNA-Free Kit (Thermofisher Scientific, USA), according to the manufacturer’s instructions. First strand cDNA synthesis was performed on DNase-treated RNA using the Superscript IV VILO Master Mix (Thermofisher Scientific, USA), according to the manufacturer’s instructions, using a Labnet International TC9610 MultiGene Optimax thermal cycler (Labnet International, USA). The resulting cDNA was diluted 1:10 using nuclease-free water to reduce the concentration of PCR inhibitors and stored in a -20°C freezer.

#### Quantitative PCR

For this experiment, we chose to use a two-gene transcriptional model in order to reduce per-sample costs associated with generating mosquito age predictions. *Aedes aegypti sarcoplasmic calcium-binding protein 1* (AAEL008844-RA, XM_001653412) and *Aedes aegypti aquaporin-11* (AAEL014255-RA, XM_001648269) had both been characterized in previous transcriptional profiling studies where they exhibited a linear decrease in expression with age [17,28,29,31]. Expression levels of those two test genes were quantified relative to the expression of the *Aedes aegypti* 40S ribosomal protein S17 gene (AAEL004175-RA, XM_001648517), a housekeeping gene that exhibits stable transcription with mosquito age [29], using previously published primers [27,28].

Quantitative PCR was performed using the PowerUp SYBR Green Master Mix (Thermofisher Scientific, USA) on a CFX96 real-time quantitative thermocycler (Bio-Rad Laboratories, USA). Each reaction contained 5 μL of PowerUp SYBR Green Master Mix, 0.5 μL each of the forward and reverse primers (stock concentrations 10mM), 2 μL of cDNA template and 2 μL of molecular water. Samples were incubated at 50°C for 2 minutes, 95°C for 2 minutes, and then forty cycles of 95°C for 15 seconds followed by 60°C for one minute. Triplicate reactions were performed for each transcript and an average Ct (cycle threshold) value was calculated for each gene, for each sample. Each plate contained a positive control, a no template control, and 6 calibrator samples (two per gene), which served as indicators of excessive plate-to-plate variation in expression. A melt-curve was performed at the end of each qPCR run (60–95°C) to confirm that amplified products for each gene corresponded to the correct melting peak.

#### Age predictions

Age predictions were generated using canonical redundancy analysis to create a calibration model from the regression of gene expression measures of known age mosquitoes (based on the method of [27]). The Ct values were log_10_-transformed in order to calculate the relative transcript abundance of the three genes in the reference colony. A non-parametric multivariate bootstrapping procedure to predict the age of field-collected mosquitoes using log contrast values (S2 File) and this was repeated for known-age mosquito specimens, to assess the predictive accuracy of the model (S3 File). Inverse regression of gene expression calibration models from the redundancy variate were used to generate age estimates for each sample. To evaluate accuracy of this calibration model, a correlation coefficient was calculated. This provided an indication of the amount of variation in gene expression explained by mosquito age [31]. The complete procedure was implemented using a script in R version 4.1.2, R Core Team 2021 [31].

Bootstrapped age predictions were rounded to the nearest whole day. Predictions that were less than one day of post-eclosion age were adjusted to a value of one. Prediction values were used to estimate the *Ae*. *aegypti* population age structure at each study location, and across the Florida Keys, as a whole. These data were also used to estimate the likely proportion of each population that had lived beyond the EIP. Since the EIP is temperature dependent, the average temperatures of our three study locations were calculated for the months of October (28.0°C), November (25.7°C), and December (21.1°C). Although no studies have specifically established the DENV EIP for Florida Keys *Ae*. *aegypti*, previously published studies indicate that the DENV EIP lasts between 6 and 15 days when temperatures are between 20°C and 30°C [42–44]. Accordingly, samples were also allocated to one of three age bins based on the likely duration of the dengue virus EIP during the collection period: pre-EIP, (mosquitoes aged between 1–5 dpe, which were likely younger than estimated EIP age range); within-EIP, (mosquitoes aged 6–14 dpe, which were likely within the estimated EIP age range); and post-EIP (mosquitoes aged 15 dpe and older that had likely survived beyond the estimated EIP and could be considered potential vectors of dengue virus) [17]. These categories were decided upon with the knowledge that the EIP is variable between mosquitoes, and those assigned to the within-EIP group may be capable of transmitting virus under certain conditions.

Population age structure data were compared between the three locations using Chi square tests for independence in GraphPad Prism 10.02, with a false discovery rate of 5% applied as a multiple test correction. Comparisons were made using raw count data and also simulated data estimating a population size of either 100 adult females per site, or with the total population size set at the total collections for each location using Microsoft Excel, with the proportionate distribute of samples per age bin from the former applied to the latter analysis.

#### Estimates of daily probability of survival

To generate estimates of the daily probability of survival (*p*) using the log-linear method, age prediction data from each location, and data combined across all locations, were binned at two-day intervals to produce counts of samples in specific age classes (S4 File). This interval allowed for a reduction in the number of zeroes in the data and produced regression coefficients and estimates of *p* that were comparable with published data [8]. Count data were transformed by adding one and taking the natural log. These data were graphed against predicted age, linear lines of best fit were fitted in Microsoft Excel and estimates of *p* were taken as the antilog of the regression coefficient of each line.

Further estimates of *p* were produced using non-linear regression, assuming a change in *p* as mosquito age increases (S5 File), as used historically with data from mark-release-recapture experiments [7]. In this context, the expected mosquitoes recaptured at time *j* (*E*_*C*_) is a function of the initial number of mosquitoes released (*N*), the recapture rate (θ), and the daily survival rate (*p*):

$$E_{C}=N\theta {\left(1-\theta \right)}^{j-1}p^{j}$$ [1]

With transcriptional profiling, no estimates of release numbers or recapture rate are made. Instead, *N* can be estimated as the mosquito population size at the collection site, and θ becomes the proportion of that population sampled to make age predictions. To that end, *j* becomes the predicted age in days, and *E*_*j*_ the number of specimens with age *j* as estimated through mosquito age predictions made via canonical redundancy analysis. Eq 1 can therefore be rearranged to estimate the daily probability of survival at a given a predicted mosquito age (*p*_*j*_) as follows:

$$p_{j}=\sqrt[{j}]{\frac{E_{j}{\left(1-\theta \right)}^{1-j}}{N\theta }}$$ [2]

For each study location, observed log_10_-transformed *E*_*j*_ data were plotted over time to determine whether the data fit the pattern of declines over time, similar to mark-release-recapture experiments [7]. To validate estimates of *N* and θ for each location and for the combined Florida Keys data, age predictions were binned to two-day intervals and *E*_*C*_ was calculated using Eq 1, using the loglinear estimate of *p* for the whole Florida Keys population. These estimates of *E*_*C*_ were compared to the observed data generated through age predictions in order to understand whether there was a difference between observed and expected data, which would indicate that *p* was unlikely to be constant with age. Data were binned to two-day intervals, as above. Next, for all values where *E*_*C*_ ≥ 0, *p*_*j*_ was estimated for each of the four data sets using Eq 2. To generate 95% confidence intervals on these estimates, age prediction data were bootstrapped 500 times in Microsoft Excel using the formula: = INDEXdata_range, RANDBETWEEN(1, ROWS(data_range)),1), with each iteration containing a random sampling of data, with replacement, at a sample size equivalent to the number of age predictions that were generated for each data set. Normality of bootstrapped *p*_*j*_ values was assessed using the D’Agostino & Pearson test in GraphPad prism. Median *p* and 95% confidence interval values were then calculated for each data set.

#### *Florida Keys* Aedes aegypti *population age structure*

To describe the structure of the Florida Keys *Ae*. *aegypti* population, age prediction data from all three locations were binned to five-day, non-overlapping intervals then converted to percentages (S6 File). These data were then compared against two simulated population age structures produced using the linear (*p*_*l*_) and non-linear (*p*_*nl*_) estimates of *p* for the entire Florida Keys population, as above. Predicted age data were also binned to the three EIP age classes, described above. Florida Keys EIP- and five-day binned data were compared against the two simulated data sets using Chi-square tests to evaluate whether there were differences between observed versus expected age structures. For these tests, sample sizes were set at 75 (the number of age predictions made), and a 5% false discovery rate was applied as a multiple test correction. For five-day binned data, older samples were combined into a single 26+ dpe bin in order to satisfy the test conditions. The 500 bootstrapped age prediction data, used to calculate *p*_*nl*_, were used to estimate the likely proportion of samples in the post-EIP age class.

## Results

### Study location parameters

The daily mean temperature for the three sites during the 2020 study period (Table 1) was 28.1°C in October (Min. 24.4, Max. 30.3°C), 25.7°C in November (Min. 23.3, Max. 30.3°C), and 21.1°C in December (Min. 18.0, Max. 24.2°C) [45]. Overall, no significant difference in temperature between locations was observed (ANOVA; *F* = 0.120, *df* = 2, *P* = 0.89). Similarly, there was no significant difference in daily rainfall between locations (ANOVA; *F* = 0.267, *df* = 2, *P* = 0.77). However, Marathon received more rain during the study period (525.5mm), than either Key West (425.2mm), or Key Largo (468.4mm).

**Table 1:** Weather conditions at the three study sites during the collection period in 2020.

	Key West		Marathon		Key Largo	
	Temp	Precip.	Temp	Precip.	Temp	Precip.
Oct	28.1	197.4	27.9	340.1	28.2	326.6
Nov	25.4	191.0	25.7	160.8	26.1	290.1
Dec	21.1	36.8	21.1	24.6	21.2	40.9

Average daily temperature (°C) and total precipitation (mm) data for each study site, aggregated from the National Weather Service (NWS, 2020). Abbreviations: Dec–December; Nov–November; Oct–October; Precip.–precipitation; Temp—temperature.

### Collection of *Aedes aegypti* samples of unknown age

Mosquito collection periods across the three locations varied due to differences in local *Ae*. *aegypti* population sizes. In Key Largo, 717 adult female specimens were collected in November 2020, with 36 specimens collected from a single site in one trapping night (November 17^th^, 2020). Populations sizes in Marathon and Key West were smaller, and collections occurred between November and December 2020. In Key West, 148 females were collected from eight sites, while in Marathon 237 females were collected from three sites. Forty females from each location were randomly selected and RNA extracted. However, only 75 of those extractions produced high quality RNA suitable for transcriptional profiling (Key Largo: *N* = 26; Marathon: *N* = 21; Key West: *N* = 28).

### Transcriptional profiling

**Fig 2:**
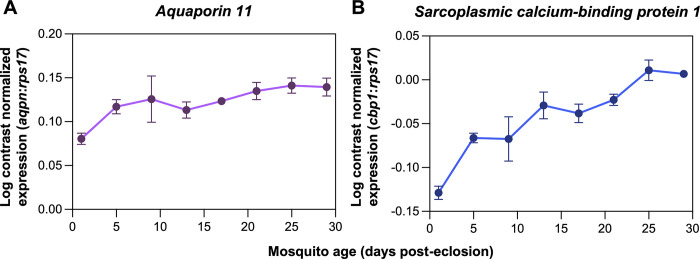
Relative transcription profiles of test genes in *Ae*. *aegypti* of known age. The expression of *Aquaporin-11* (*aqpn*), putative (XM_001648269) **(A)**, and *Sarcoplasmic calcium-binding protein 1* (*cbp1*), putative (XM_00653412) **(B)** was calculated for cohorts of *Ae*. *aegypti* females with known ages between 1 and 29 days post-eclosion using RT-qPCR. Log contrasts were derived by calculating the log_10_ of the ratio of each gene to the reference gene, *40S ribosomal protein 17* (XM_0016485147). Upward trends in log contrast normalized expression indicate decreasing transcript abundance with age. Each data point represents the mean log contrast normalized expression ± sem. Sample size varied between 3 and 12 specimens per age group.

**Fig 3:**
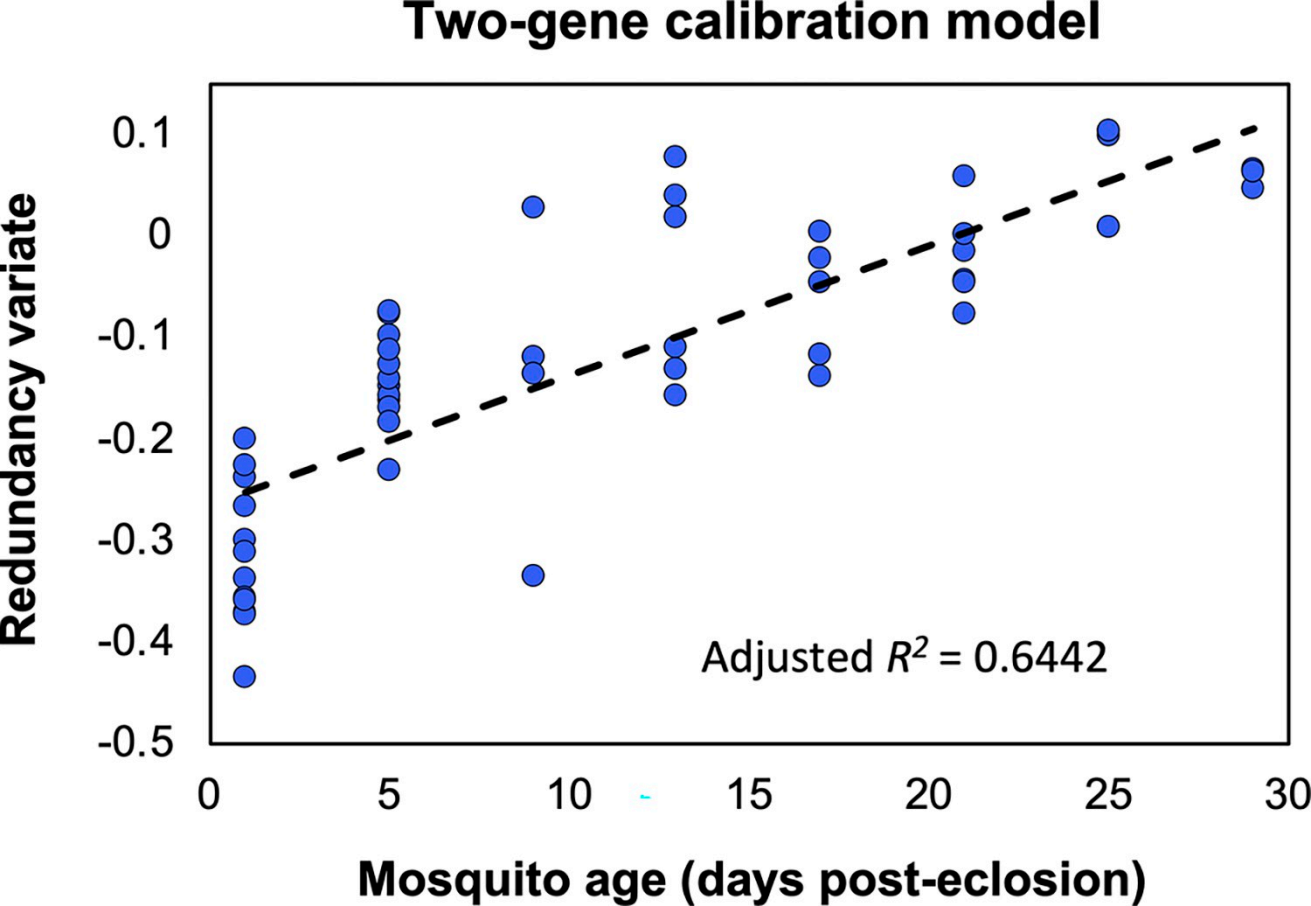
Two-gene calibration model based on known age Florida Keys *Ae*. *aegypti*. Canonical redundancy analysis was used to convert log contrast normalized gene expression data into redundancy variate data for each known age mosquito specimen (blue dots). An adjusted *R*^*2*^ value of 0.6442 suggested there was a strong relationship between redundancy variates and mosquito age, making the model suitable for use in making age predictions. The dashed line indicates the linear line of best fit for the data.

All primer sets effectively amplified gene targets for Florida Keys *Ae*. *aegypti* despite being designed for *Ae*. *aegypti* populations from Australia. The expression profiles for both *aquaporin-11* and *sarcoplasmic calcium-binding protein 1* demonstrated a linear decrease in expression with known age samples (Fig 2), similar to what was observed in previous studies from Australia [28] and Vietnam [31]. The known age calibration model generated through canonical redundancy analysis (Fig 3) had a coefficient of determination of 0.64 (*N* = 51, *R*^*2*^ = 0.644, *P* < 0.0001), while the correlation of the redundancy model was 0.80, with both values indicating that there was a strong overall correlation of the redundancy variate with variation in mosquito age.

### Predictive accuracy of the model

The known age prediction model was validated by generating age predictions for each known-age specimen and then calculating differences between their actual and predicted age (Table 2). For the entire cohort of known-age mosquitoes, the mean predictive accuracy was ± 5.65 days (± 0.57 days, s.e.m.) of actual age. Predictive accuracy was then assessed for each of the three age groups: pre-EIP (1–5 days dpe) (*N* = 14), within-EIP (6–14 dpe) (*N* = 18), and post-EIP (15+ dpe) (*N* = 19). We observed a significant difference in accuracy across the three age groups (ANOVA; *F* = 156.3, *df* = 2, *P* < 0.0001), with the lowest accuracy for the pre-EIP group and the highest accuracy for the within-EIP group. A sharp decrease in the expression of both test genes between 1- and 5-dpe led to a number of negative age predictions for known-age (*N* = 9) and unknown-age cohorts (*N* = 14), which were adjusted to an age of 1 dpe.

**Table 2:** Predictive accuracy of models by mosquito age group.

Mosquito Age Group (Days post-eclosion)	*N*	Mean predictive accuracy (± sem)
Pre-EIP (1–5)	14	6.96 (± 1.29)
Within-EIP (6–14)	18	4.70 (± 0.62)
Post-EIP (15+)	19	5.59 (± 1.04)
All Samples	51	5.65 (± 0.57)

Data are presented as mean difference in days (± sem) between predicted and actual age.

Abbreviations: EIP—extrinsic incubation period. *N*—number of samples. sem—standard error of the mean.

### Age predictions of field specimens

**Fig 4:**
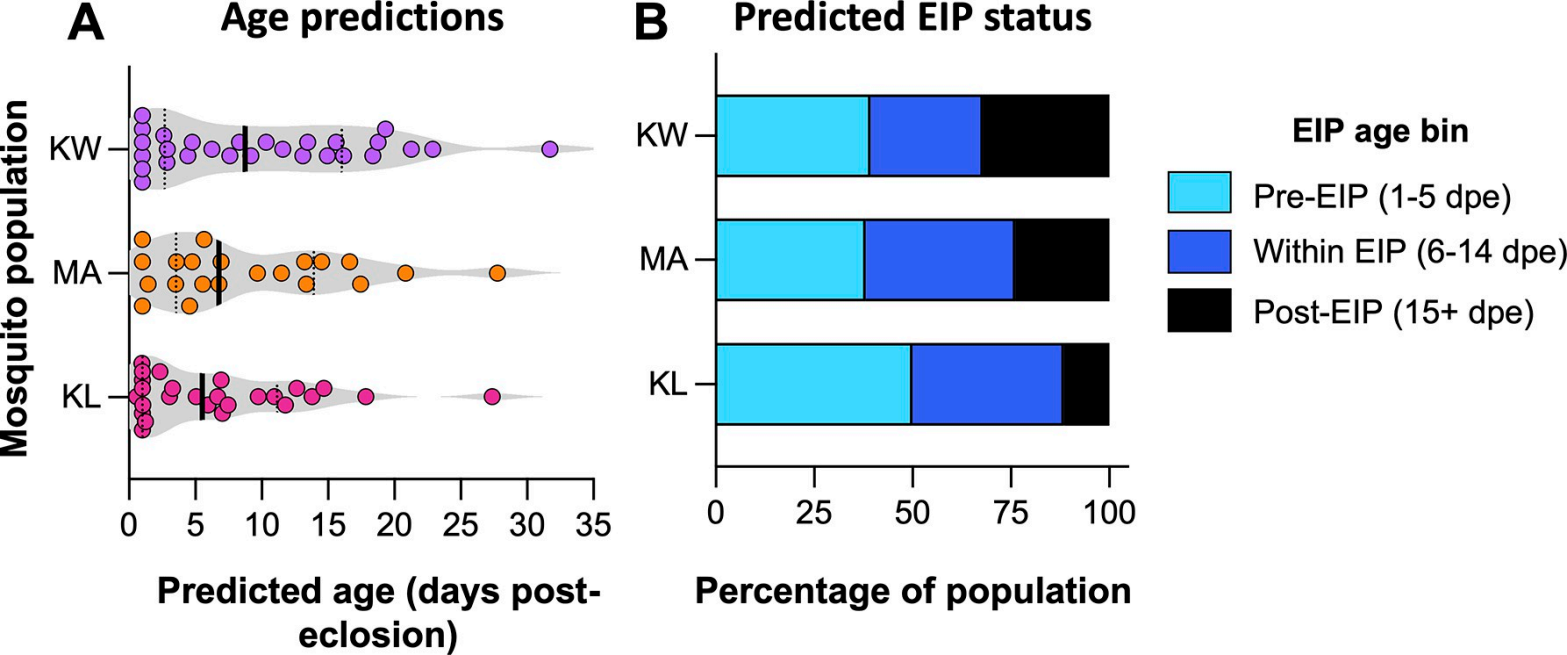
Predicted ages and estimated *Ae*. *aegypti* population age structures from three locations in the Florida Keys. Violin plots of predicted ages (days post-eclosion) of field-derived, adult, female *Ae*. *aegypti* **(A)**, collected from Key West (KW, *N* = 28), Marathon (MA, *N* = 21), and Key Largo (KL, *N* = 26). Predictions were generated via non-parametric multivariate bootstrapping. Colored dots indicate values of age predictions for individual specimens. Solid, black lines represent group medians. Dashed black lines indicate group interquartile ranges. Age predictions were binned into three age categories relevant to the DENV EIP (Pre-EIP (predicted age 1–5 dpe), Within EIP (6–14 dpe), and Post-EIP (15+ dpe)) to create a stacked bar plot depicting potential EIP status of Florida Keys *Ae*. *aegypti* populations **(B)**. Data indicated that Key West supported the oldest population, with the greatest proportion of specimens in the Post-EIP age bin. Meanwhile, Key Largo had the youngest population with 50% of specimens yielding age predictions of 5 dpe or younger.

Canonical redundancy analysis produced median age predictions of 5.51, 6.76, and 8.75 dpe for Key Largo, Marathon, and Key West, respectively (Fig 4A), suggesting that Key Largo had the youngest *Ae*. *aegypti* population and Key West the oldest. After allocation of predicted age data to the three EIP age bins (1–5 dpe, 6–14 dpe, 15+ dpe), there was no significant difference in the population age structure between the three locations (Fig 4B; Chi Square test; *X*^*2*^ = 3.53, *df* = 4, *N* = 75, *P* = 0.4738). The size of the post-EIP bin was 11.54% in Key Largo, 23.81% in Marathon, and 32.14% in Key West.

### Estimates of daily survival rate

**Fig 5:**
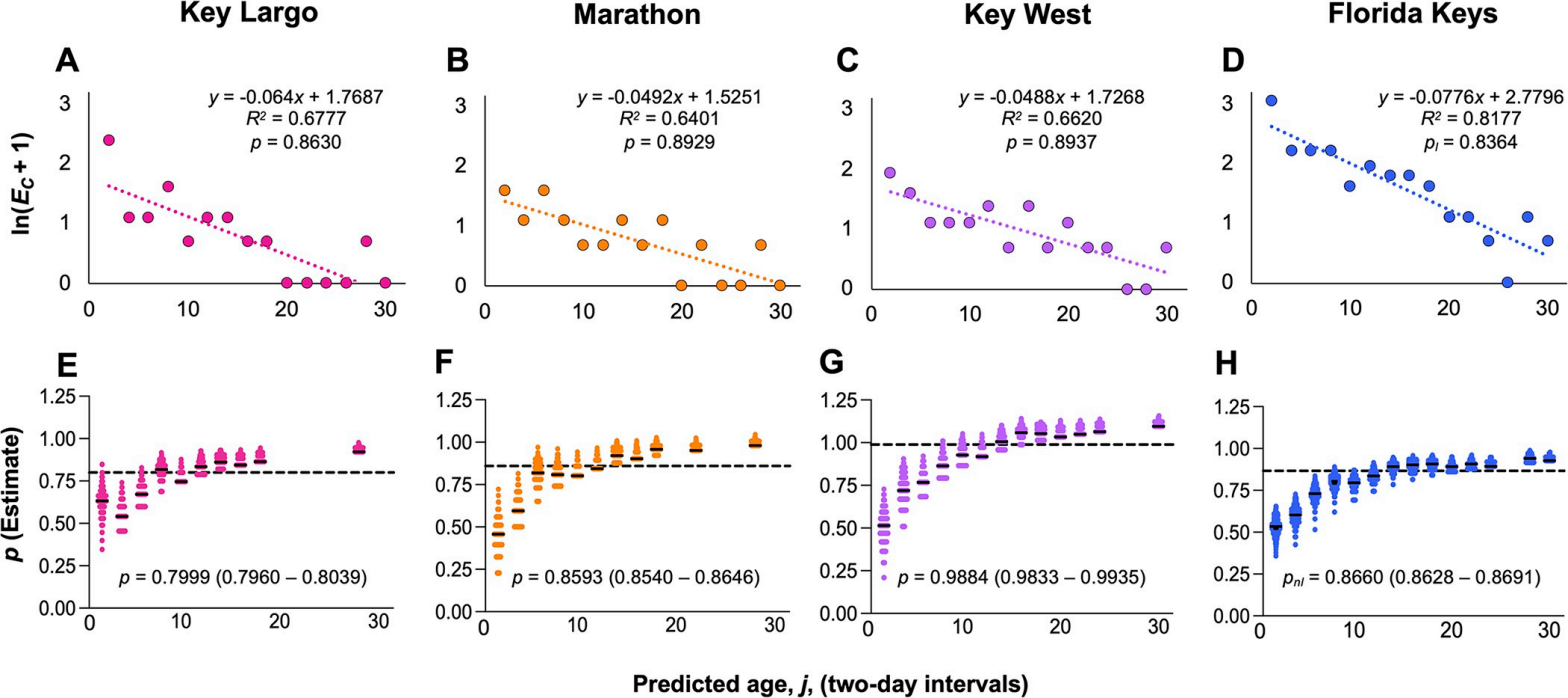
Estimates of daily survival rate, *p*, for Florida Keys *Ae*. *aegypti* populations. Loglinear plots of sample counts versus mosquito age were used to generate linear estimates of *p* for *Ae*. *aegypti* populations from Key Largo **(A)**, Marathon **(B)**, Key West **(C)**, and for the Florida Keys as a whole **(D)**. Sample counts from two-day age bins were transformed using the equation *y* = ln (*x* + 1). Estimates of *p* were taken as the antilog of the regression coefficient of linear lines of best fit (dotted lines) and were greater than *p* = 0.8000 for all data sets. Non-linear regression was used to generate further estimates of *p* for Key Largo **(E)**, Marathon **(F)**, Key West **(G)**, and for the Florida Keys **(H)**. Estimates were generated at two-day intervals based on sample counts per age bin, as determined via age predictions, as well as the estimated population size and sampling rate. Median *p* values and 95% confidence intervals for each population were generated via bootstrapping with replacement. Data on dot plots indicate bootstrapped estimates of *p* for mosquitoes of a given age. Black dashed lines indicate the median of each data set. Solid black lines indicate median *p* values for each age bin. All data sets displayed an increase in the value of *p* with mosquito age. Linear (*p*_*l*_) and non-linear (*p*_*nl*_) estimates of *p* for the Florida Keys were used to estimate the age structure of the *Ae*. *aegypti* population in the region.

Loglinear estimates of the daily probability of survival (*p*) for *Ae*. *aegypti* in the Florida Keys were generated for each location (Fig 5A–5C). Through this method, estimates of *p* were greatest for Key West (*p* = 0.8937), slightly lower in Marathon (0.8929), and lowest in Key Largo (0.8630). The estimate of *p* for the Florida Keys as a whole (*p*_*l*_) was 0.8364 (Fig 5D). All data sets showed strong, linear, decreasing relationships between predicted mosquito age and number of specimens per age bin (*R*^*2*^ ranging from 0.6401 to 0.8177).

To generate estimates of *p* through non-linear regression, the predicted population size at each site (*N*) was taken as the number of specimens collected during the collection period. The proportion of mosquitoes sampled per location, θ, was estimated as 0.0362 for Key Largo (26/717 specimens sampled), 0.0886 for Marathon (21/237), and 0.1892 for Key West (28/148). Log_10_-transformed specimen count data across age bins revealed a strong non-linear relationship between counts and predicted age (S1 Fig). Evaluation of Eq 1, which estimated *E*_*j*_, specimen counts at age *j*, given a constant value of *p* = 0.8364, revealed that fewer specimens than expected were observed in the younger age bins, while more specimens than expected were observed in older age bins (S2 Fig). This pattern was consistent across the four data sets.

Through Eq 2, bootstrapped estimates of *p* were determined to be lower for younger mosquitoes and then higher amongst older mosquitoes, indicating that *p* was not constant with age (Fig 5E–5H). For the three locations, median *p* was estimated to be 0.7999 for Key Largo (Number of estimates (*N*_*est*_) *=* 3995; C.I._0.05_ = 0.7960–0.8039), 0.8593 for Marathon (*N*_*est*_ *=* 4339; C.I._0.05_ = 0.8540–0.8646), and 0.9884 for Key West (*N*_*est*_ *=* 5243; C.I._0.05_ = 0.9833–0.9935). Estimates of *p* for Key West were likely inflated due to the small sample size, a high sampling rate, and a far greater than expected number of specimens predicted to have an age in excess of 15 dpe. For the Keys as a whole, the median estimate of *p* using this method *p*_*nl*_, was 0.8660 (*N*_*est*_ *=* 6460; C.I._0.05_ = 0.8628–0.8691). Interestingly, median *p* was 0.7444 for mosquitoes with a predicted age between 1–12 dpe (*N*_*est*_ *=* 2990; C.I._0.05_ = 0.7402–0.7486), compared to 0.9082 for those with a predicted age of >14dpe (*N*_*est*_ *=* 3470; C.I._0.05_ = 0.9072–0.9092).

### Predicted population age structure of *Aedes aegypti* in the Florida Keys

**Fig 6:**
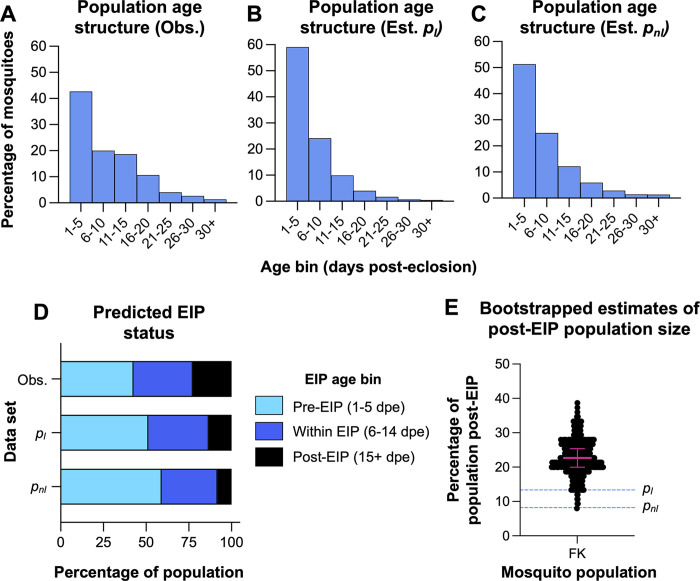
Predicted age structure of Florida Keys *Ae*. *aegypti* population. Models of the population age structure of Florida Keys *Ae*. *aegypti* were generated with five-day age bins. Observed data **(A)** were compiled based on the age predictions generated through transcriptional profiling. Comparison with estimated population age structures based on *p*_*l*_ = 0.8364 **(B)** and *p*_*nl*_ = 0.8660 **(C)** indicated that the observed age structure had fewer younger mosquitoes and more older mosquitoes than expected. This was corroborated when looking at potential EIP status **(D)**, with the observed population having a significantly greater proportion of post-EIP mosquitoes. Bootstrapped estimation of the size of the post-EIP population in the Florida Keys **(E)** yielded a median value of 32.14% of the population, a value which exceeded the predicted values by approximately 20%. In panel E, black dots depict individual estimates of the size of the post-EIP population generated for bootstrapped data. Pink lines depict the median estimate ± 95% confidence interval. Dashed, blue lines depict the estimates of the post-EIP population size predicted from *p*_*l*_ and *p*_*nl*_.

The observed age structure of the Florida Keys *Ae*. *aegypti* population (Fig 6A), was compared to hypothetical population age structures of populations experiencing the linear (Fig 6B, *p*_*l*_ = 0.8364) and non-linear (Fig 6C, *p*_*nl*_ = 0.8660) estimates of *p*, computed above. With data divided into five-day intervals, the observed population age structure was significantly different from one predicted by *p*_*l*_ at *N* = 250 (Chi Square test; *X*^*2*^ = 20.52, *df* = 5, *P* = 0.001) and *N* = 500 (Chi Square test; *X*^*2*^ = 57.93, *df* = 5, *P* < 0.0001). It was also significantly different to the age structure predicted by *p*_*nl*_ at *N* = 500 only (Chi Square test; *X*^*2*^ = 22.03, *df* = 5, *P* = 0.0005). Comparison of EIP binned age structures for the observed Florida Keys data, *p*_*l*_, and *p*_*nl*_, revealed a greater proportion of post-EIP mosquitoes in the observed dataset compared to what was expected by the estimated distributions (Fig 6D). Comparison of observed and *p*_*l*_ age structures revealed significant differences at all four sample sizes tested (Chi Square test, *df* = 2; *N* = 75, *X*^*2*^ = 7.175, *P* = 0.00277; *N* = 100, *X*^*2*^ = 9.102, *P* = 0.00106; *N* = 250, *X*^*2*^ = 22.73, *P* < 0.0001; *N* = 500, *X*^*2*^ = 47.14, *P* < 0.0001). Meanwhile, comparison of observed and *p*_*nl*_ age structures revealed significant differences at *N* = 250 (Chi Square test; *X*^*2*^ = 7.217, *df* = 2, *P* = 0.0271) and *N* = 500 (Chi Square test; *X*^*2*^ = 15.92, *df* = 5, *P* = 0.0003). Collectively, these observations suggested that *p*_*nl*_ was a better fit for the observed data than *p*_*l*_. Overall, observed data had fewer younger mosquitoes and more older mosquitoes than expected. Bootstrapped estimates of the percentage of the Florida Keys *Ae*. *aegypti* population in the post-EIP bin (Fig 6E) revealed a median value of 32.14% (N.O.E. *=* 500; C.I._0.05_ = 31.34–32.95%), which exceeded the values predicted by *p*_*l*_ (8.20%) and *p*_*nl*_ (13.33%), and the observed value (22.67%).

## Discussion

In this study we demonstrate that a two-gene transcriptional model can be used to generate age predictions of *Ae*. *aegypti* mosquitoes from the Florida Keys. Our data suggest that *Ae*. *aegypti* population age structures are similar across the Keys, but hint that age of the Key West population may be somewhat older and the age of the Key Largo population slightly younger. We demonstrate that age predictions can be used to generate estimates of daily survival rate, *p*, that are comparable to the literature. These estimates reveal that the Florida Keys likely supports a large population of older, post-EIP mosquitoes, potentially facilitating dengue transmission in the region. However, our analysis also suggests that the size of that post-EIP population might be overestimated in our data.

### Age grading via transcriptional profiling for *Aedes aegypti* in the Florida Keys

Our data suggest that the transcriptional profiling method of age prediction used with mosquito populations in other regions of the world is suitable for use with *Ae*. *aegypti* in the Florida Keys, an important site for dengue virus transmission in the Unites States [17,28,29,31]. Our model had similar limitations to those from previous studies. For instance, mean predictive accuracy (± 5.65 days) was comparable to that of past models where accuracy ranged from ± 3.97 days to ± 7.04 days [28,31]. We observed a greater degree of age-dependent change in *sarcoplasmic calcium-binding protein 1* compared to *aquaporin-11*, suggesting that the former could be used as the basis of a one-gene age prediction model, in order to reduce per-sample costs. We also observed a strong, linear change in the redundancy variate with age, but this weakened in older mosquitoes where expression of the target genes was less prone to change with age [28,31]. This could potentially be mediated by making more frequent measurements of gene expression amongst older mosquitoes in the known age cohort.

Interestingly, our model was most accurate for mosquitoes within the EIP, but less accurate for younger and older, post-EIP mosquitoes, a recurring limitation of the approach [31]. These limitations suggest that there may be issues in accurately differentiating post-EIP mosquitoes, but also that these transcriptional models satisfactorily discriminate mosquitoes that are likely old enough to transmit dengue from those that are not. That being said, an EIP of 6–14 days and a mean accuracy of ± 5.65 days will likely lead to some erroneous determination of age and potential vector status. It should be noted that EIPs are not absolute, and there are host, virus, and environmental factors associated with variation in EIP duration [46–49]. Our selected ages defining pre-, within-, and post-EIP categories may not always reflect the real age at which *Ae*. *aegypti* mosquitoes become capable of spreading dengue virus in nature.

In Florida, peak season for *Ae*. *aegypti* and arbovirus transmission occurs during mid-summer when environmental conditions are typically warm and wet. However, our study ran during the cooler, drier part of the year when conditions are less favorable for *Ae*. *aegypti*. Depending on the impact of seasonality, environmental variability could feasibly limit the applicability of our data to mosquito populations in the summer. Gene expression in mosquitoes varies depending on the ambient temperature experienced by the host [50–52]. Likewise, transcriptional profiling-based age grading is subject to environmental variation. The expression of the calcium-binding protein is temperature dependent, with decreased expression in older mosquitoes exposed to high temperature [32]. Changes in ambient temperature can also impact the predictive accuracy of age grading models [33]. Furthermore, less accurate age predictions have been observed in semi-field studies compared to laboratory studies, likely driven by exposure to a more complex environment [28,33]. Finally, there is an impact of seasonality, with marked decreases in accuracy seen during the wet/cool season in Vietnam [31]. As such, it is feasible that the estimates of *Ae*. *aegypti* age, population age structure, and daily probability of survival made here may not be fully applicable to other seasons.

### Daily survival of *Aedes aegypti* in the Florida Keys

Our analysis suggested that daily survival rates (*p*) for *Ae*. *aegypti* in the Florida Keys were high in all three locations, with a maximum estimated daily mortality rate of just over 20%. Our estimates of *p* ranged between 0.8364 and 0.8937 when calculated via the loglinear method, and between 0.7999 and 0.9884 using non-linear methods. For the Florida Keys as a whole, our data suggest that *Ae*. *aegypti* experience a daily mortality rate of between 13.34% (non-linear method) and 16.36% (linear method). These values are in line with previous estimates of daily survival, with historical estimates from the literature varying between 0.68 and 0.98, depending on the species and study [6]. For instance, 0.607–0.779 were estimated for *Ae*. *aegypti* in Rio de Janeiro, Brazil [53,54], 0.897 for *Ae*. *aegypti* in Vietnam [31], 0.890 for *Anopheles sinensis* in South Korea [55], 0.82 for *Anopheles arabiensis* [56], and 0.91 for *Anopheles gambiae* [57].

We observed that mortality rates were the highest in Key Largo and lower in Marathon and Key West. Estimates of *p* across locations were more variable when calculated via the non-linear method than via the linear method. It is common for estimates of *p* to vary depending on the method of calculation [7,53,54]. However, it is possible that our non-linear estimates of *p* were underestimated for Key Largo and overestimated for Key West as this method is dependent on the number of samples or, in this instance, the number of age predictions. Prediction numbers were low at individual locations, and data were skewed at Key Largo and Key West, which contained a disproportionate number of younger and older individuals, respectively. The latter location was also oversampled in comparison to Marathon and Key Largo, which could have influenced the estimate. As such, we can’t discount the prospect that our estimates of *p* were subject to errors of precision [58]. Nevertheless, if consistent and valid, our data potentially suggest that the mortality risk faced by *Ae*. *aegypti* might vary across the Florida Keys. Such differences could potentially arise due to ecological, socioeconomical, or entomological heterogeneities, or differences in mosquito control activities between the areas. The very high density and the older populations *of Ae*. *aegypti* in Key West observed (Fig 4) in this study may be the product of an inherently conducive environment for the reproduction and survival of this vector mosquito.

All four of our non-linear models used to estimate *p* indicated that survival rate of *Ae*. *aegypti* in the Florida Keys varied with age, in line with previous studies [7–9]. In our data, we observed high mortality rates for younger mosquitoes, which declined amongst older mosquitoes. Slowing of mortality in older individuals has been observed in insect populations [59,60]. In contrast, mark-release-recapture studies of *Ae*. *aegypti* in Florida and Thailand comparing released cohorts of different ages revealed that *p* either decreased with age or did not change with age when calculated via non-linear methods but increased for older mosquitoes when calculated using log-linear methods [8]. Furthermore, in a large-scale experiment, *Ae*. *aegypti* mortality was observed to be low in young mosquitoes, then accelerated with age, before eventually declining for very old mosquitoes [9]. Such disparities may have arisen due to the use of different mortality functions. Additionally, our analyses (S1 and S2 Figs) indicate that our data likely underestimate the number of younger mosquitoes and overestimate the number of older mosquitoes in each population, which may have contributed to our observation of declining mortality rates in older mosquitoes. However, low, stable mortality amongst older *Ae*. *aegypti* in the Florida Keys, if it does occur, might contribute to dengue transmission in the region.

### *Aedes aegypti* population age structure and dengue risk in the Florida Keys

Population age structure is an important element of vectorial capacity in *Ae*. *aegypti* as mosquitoes must survive beyond the EIP to be able to transmit a pathogen. Modelled population age structures for Key West, Marathon, and Key Largo indicate that each site is capable of supporting large numbers of older mosquitoes that are potentially capable of transmitting dengue virus. Local outbreaks have been reported in both Key Largo [36] and Key West [35] but, at the time of writing, not in Marathon, even though mosquitoes from these three locations exhibited similar human blood feeding rates [61]. Our data suggest that each location is likely capable of supporting local dengue transmission. For Key West, a very high proportion of post-EIP mosquitoes represents a major risk factor. For Key Largo, although the predicted population age structure was the youngest, 11% of mosquitoes still had post-EIP status, and the size of the local *Ae*. *aegypti* population was far larger than in the other two locations, meaning there could be a greater number of post-EIP mosquitoes, overall. Our data also indicate that it is worthwhile monitoring Marathon for local dengue transmission in the future.

Our estimates of population age structure for *Ae*. *aegypti* suggest that 22.67% of mosquitoes across the Florida Keys were aged at greater than 15 dpe. Assessments of mosquito population age structure in the modern literature have produced low but variable estimates of the post-EIP population size. For instance, post-EIP mosquitoes accounted for less than 5% of *An*. *arabiensis* and *An*. *gambiae* colony mosquito populations [24], less than 5% for *An*. *arabiensis* from Tanzania [62], less than 10% for *An*. *coluzzii* from Burkina Faso [62], and 9% for *Ae*. *aegypti* from Arizona [17]. A study of *Ae*. *aegypti* in Vietnam indicated that the size of the post-EIP population varied by as much as 3.17 times across seasons, with 92% surviving to 12 dpe during the dry/cool season compared to 29% in the wet/cool season [31]. Our estimate exceeds those of most of those studies but falls within the range of values outlined in the latter study.

Age structures based on our two estimates of *p* for the Florida Keys, *p*_*l*_ and *p*_*nl*_, indicate that we observed more older, post-EIP mosquitoes than was expected. Additionally, bootstrapped estimates of the post-EIP population size revealed a median estimate of 32.14%, which exceeded the predicted value. Indeed, 42.6% of the bootstrapped estimates exceeded our observed value of 22.67%, suggesting the possible influence of survivorship bias, with the bootstrapping process oversampling mosquitoes with older predicted ages. This disparity may have been a product of the two-gene calibration model, where redundancy variates were fairly similar across older ages (15–29 dpe), and this could have led to less precise predictions of age in older mosquitoes. Alternatively, it is possible that our observed data have underestimated the actual value of *p* in the Florida Keys. If greater than one in five female *Ae*. *aegypti* in the Florida Keys do survive beyond the dengue virus EIP during the dry season, this clearly represents a high risk factor for dengue outbreaks in the region.

### Caveats

There are some implicit biases in our sample collection and data structure that may limit the overall applicability of our findings. First, was a relatively low number of unknown age mosquito specimens collected at each site, which eventuated due to lower than expected numbers of mosquito samples with sufficiently high RNA yield. Interestingly, we observed lower RNA quality associated with specimens from Marathon, but no effects associated with time of collection. Sample size effects could have impacted our estimates of population age structures and resulting analyses. Similarly, there was a bias in our Key Largo collections, which were collected from a single trap in one night, limiting our ability to generate a representative population age structure *Ae*. *aegypti* across that site, and potentially inviting biases associated due to localized age distortions–our data did depict Key Largo as having a higher number of younger mosquitoes than the other two sites. In transcriptional profiling-based age grading, high per sample costs and the need to time match the known and unknown age specimens make it difficult to generate more than a snapshot of mosquito population age structure. Making certain that the snapshot is representative of the local mosquito population is vital. However, in spite of the costs, future studies should consider the benefits that might arise from assessing changes to population age structure and daily survival across time or between seasons.

### Conclusions and future directions

Through age grading via transcriptional profiling, we reveal that *Ae*. *aegypti* populations in Key Largo, Marathon, and Key West, three locations in the Florida Keys, all harbor large numbers of older mosquitoes that survive the extrinsic incubation period of dengue virus, enabling local transmission of the virus. Our data revealed that the Key Largo population was the largest but also the youngest, while Key West had the highest proportion of mosquitoes that had survived beyond the dengue virus EIP, potentially revealing key differences between the two populations that merit further investigation. For the Florida Keys, as a whole, we observed a daily mortality rate of between 13 and 16%, meaning that approximately 20% of mosquitoes likely survive to be old enough to transmit dengue virus, given an EIP of 14 days. Collectively, our data highlight the suitability of the Florida Keys to harbor older *Ae*. *aegypti* mosquito populations and will likely facilitate further assessment of vectorial capacity for dengue virus in the region.

## Supporting information

S1 Fig Linearity of relationship between age prediction counts and predicted age. Counts of age predictions (*E*_*C*_) for two-day age binned data were LOG_10_ (*E*_*C*_ +1) transformed and plotted against predicted mosquito age, *j*, for each of the four data sets (Key Largo, Marathon, Key West, Florida Keys). All data sets demonstrated a strong, non-linear decrease in the sample count as predicted age increased, indicating suitability of the data for assessment using Eq 2.
(TIF)

S2 Fig Observed versus expected sample count values, given a constant daily survival rate, *p*_*l*_. Estimates of *E*_*C*_ were obtained for Key Largo, Marathon, Key West, Florida Keys using Eq 1, assuming that each population was subject to a daily survival rate of *p*_*l*_ = 0.8364, with sampling rates and population sizes estimated based on mosquito collection data from this project. These estimates (black circles) were plotted against observed counts generated via age prediction (colored circles). Logarithmic lines of best fit were generated for expected (*y*_*E*_) and observed (*y*_*O*_) data for each population. These lines indicated that there fewer than expected mosquitoes of young age, but more older mosquitoes than expected in each of the four observed data sets.
(TIF)

S1 File *Aedes aegypti* field collection data. (XLSX)

S2 File Age predictions for field-collected specimens. (XLSX)

S3 File Predictive accuracy of age grading model. (XLSX)

S4 File Linear estimates of daily survival. (XLSX)

S5 File Non-linear estimates of daily survival. (XLSX)

S6 File Population age structure estimates. (XLSX)
