# Supplementary figures and images for: Estimation of population age structure, daily survival rates, and potential to support dengue virus transmission for Florida Keys Aedes aegypti via transcriptional profiling
Source: PLoS Negl Trop Dis. 2024 Aug 13;18(8):e0012350. doi: 10.1371/journal.pntd.0012350 (PMC11321583; doi:10.1371/journal.pntd.0012350)

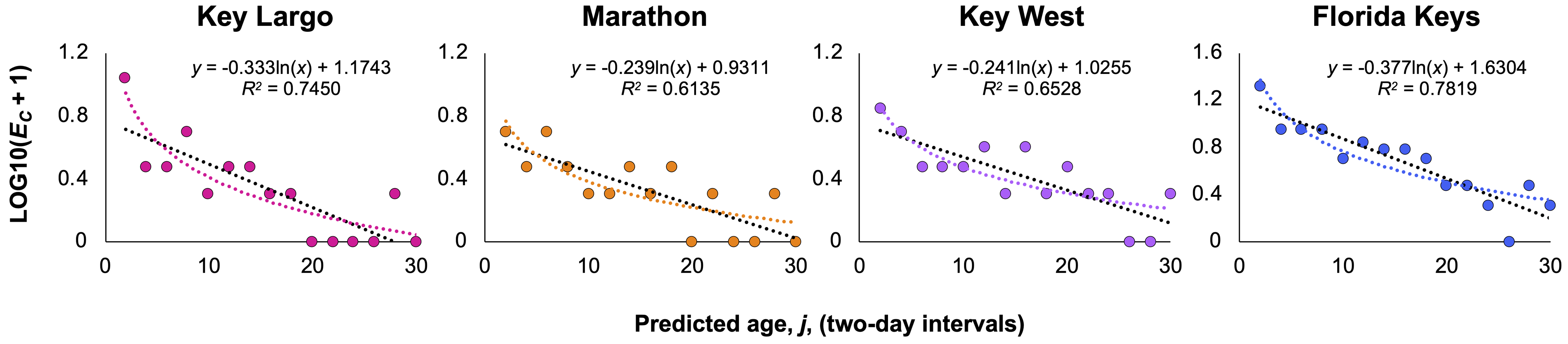

Supplement: S1 Fig — Counts of age predictions (EC) for two-day age binned data were LOG10 (EC +1) transformed and plotted against predicted mosquito age, j, for each of the four data sets (Key Largo, Marathon, Key West, Florida Keys). All data sets demonstrated a strong, non-linear decrease in the sample count as predicted age increased, indicating suitability of the data for assessment using Eq 2. (TIF) [file pntd.0012350.s001.tif]

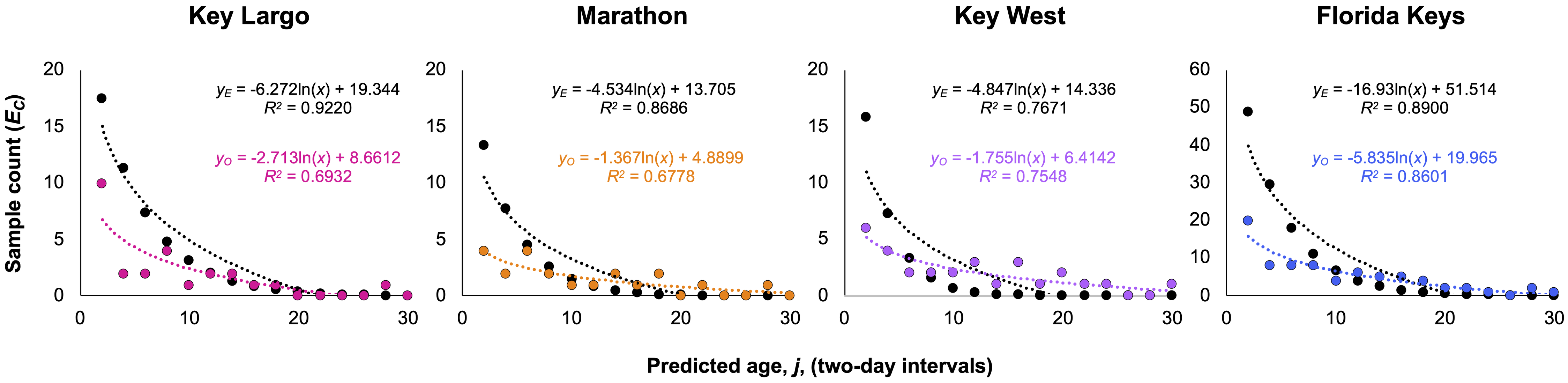

Supplement: S2 Fig — Estimates of EC were obtained for Key Largo, Marathon, Key West, Florida Keys using Eq 1, assuming that each population was subject to a daily survival rate of pl = 0.8364, with sampling rates and population sizes estimated based on mosquito collection data from this project. These estimates (black circles) were plotted against observed counts generated via age prediction (colored circles). Logarithmic lines of best fit were generated for expected (yE) and observed (yO) data for each population. These lines indicated that there fewer than expected mosquitoes of young age, but more older mosquitoes than expected in each of the four observed data sets. (TIF) [file pntd.0012350.s002.tif]
